# Vertebrobasilar Dolichoectasia and Basilar Artery Dissection Presenting With Trigeminal Neuralgia: A Case Report

**DOI:** 10.3389/fneur.2019.00491

**Published:** 2019-05-15

**Authors:** Yuhan Wang, Wenchao Cheng, Yajun Lian

**Affiliations:** Department of Neurology, The First Affiliated Hospital of Zhengzhou University, Zhengzhou, China

**Keywords:** vertebrobasilar dolichoectasia, trigeminal neuralgia, trigeminal nerve, basilar artery dissection, facial pain

## Abstract

Trigeminal neuralgia secondary to vertebrobasilar dolichoectasia and basilar artery dissection is rare. The authors report the case of a 72-year-old man with a 5-year history of right electrical facial pain identical with trigeminal neuralgia. Finally, magnetic resonance imaging and digital subtraction angiography revealed basilar artery dissection and vertebrobasilar dolichoectasia. The patient underwent partial basilar dissecting aneurysm embolization. The facial pain was relieved immediately after the operation and disappeared completely 6 months later. Three years after surgery, the patient had experienced no recurrence of the right facial pain.

## Introduction

Trigeminal neuralgia (TN) is a frequent disorder with paroxysmal hemifacial pain affecting the dermatomal distribution of the trigeminal nerve, with an annual incidence of 27 per 100,000 (1). It is often associated with the vascular compression of the trigeminal nerve root entry zone, and also related to the superior cerebellar artery or anterior inferior cerebellar artery (2). However, the direct compression by vertebrobasilar dolichoectasia (VBD) is a much less common cause of TN with an estimated general incidence of approximately 1% (3). In most cervicocerebral artery dissection cases, headache and neck pain may be the initial symptom before the ischemic stroke (4), but the TN-like facial pain is less common.

## Case Presentation

### History, Examination, and Operation

A 72-year-old man presented with a 5-year history of paroxysmal, severe and electrical right facial pain in V2 and V3 trigeminal distributions, and complained of typical tic douloureux. Episodes of pain were triggered by washing face and brushing teeth with cold water. It was not relieved by over-the-counter medications. In the third year of the disease, magnetic resonance angiogram (MRA) showed vertebrobasilar dolichoectasia for this patient, and he underwent microvascular decompression (MVD) surgery. His facial pain completely resolved immediately postoperatively but had recurred at 5 months after surgery. Then he accepted the treatment of carbamazepine (600 mg per day) and gabapentin (900 mg per day), without complete pain relief, and the pain resumed every time when the treatment was reduced. The patient reported no alalia and visual changes, no numbness, or paralysis.

He was a patient with arterial hypertension (treated with indapamide), but without diabetes and any history of trauma, tumor or multiple sclerosis–related TN. He was a former smoker and drinker (40 pack-years). Physical examination revealed a well-developed, anxious male. The neurological examination revealed hypoesthesia and hypoalgesia in right V2 and V3 trigeminal distributions, and the right eye fissure was smaller than the left. The other vital signs and physical examination were normal.

Laboratory tests documented mild anemia (red blood cell count 4.22 × 10^∧^12/L, hemoglobin 124 g/L), hypokalemia (serum potassium level 3.44 mmol/L) with normal renal function. A slight reduction in above indicators may be associated with the patient's anxiety and poor diet due to the facial pain.

**Figure 1:**
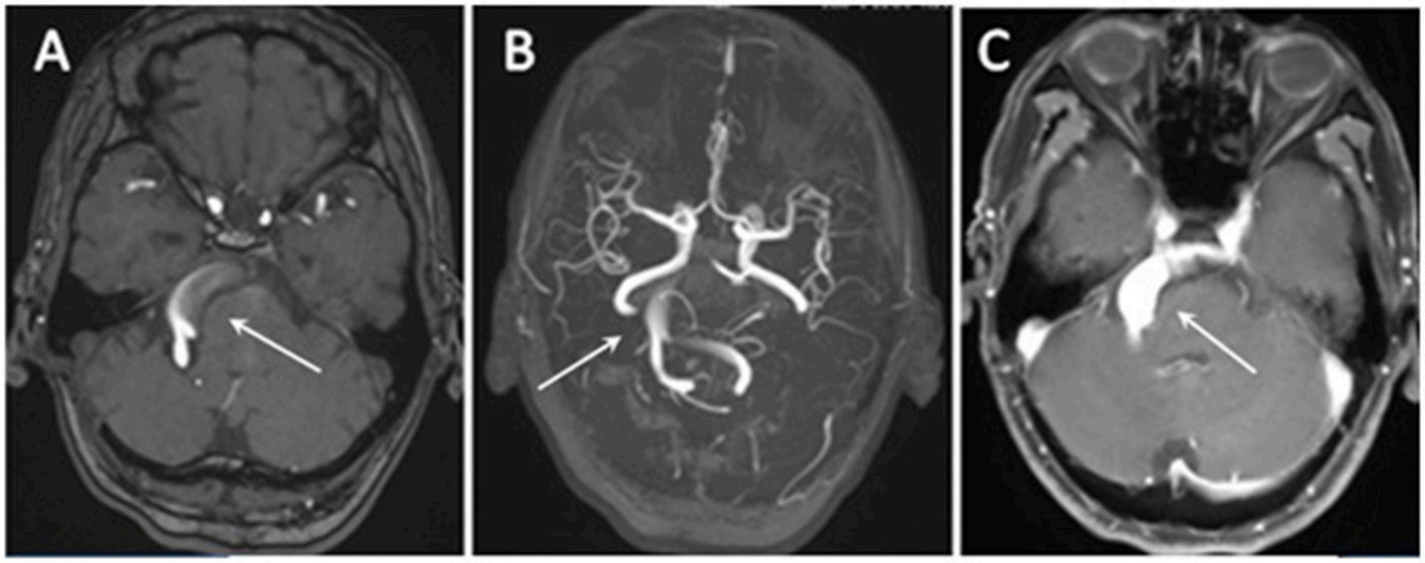
**(A,B)** 3D time-of-flight magnetic resonance angiography (TOF-MRA) revealed a dilated, tortuous, and elongated vertebral artery and basilar artery (arrow). **(C)** The contrast-enhanced MRI revealed a significant expansion of the basilar artery (arrow).

**Figure 2:**
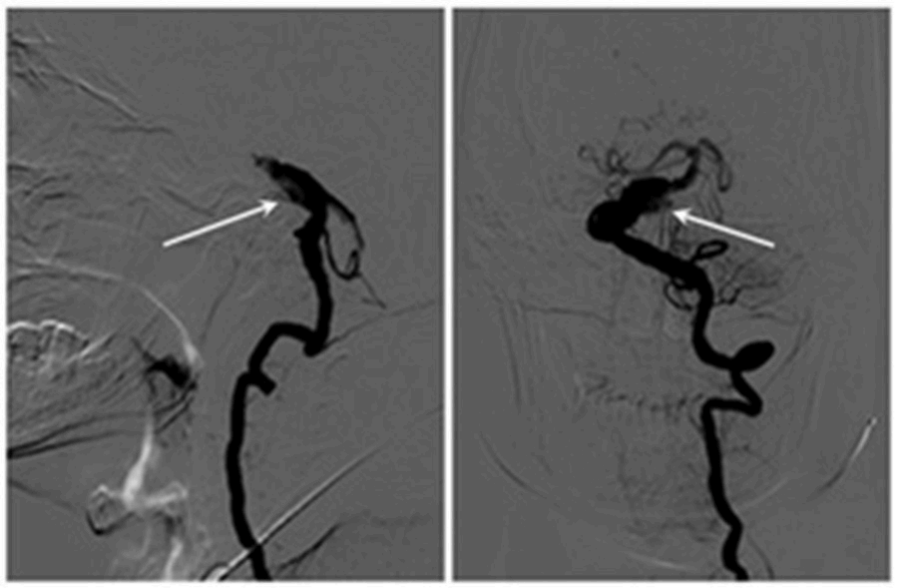
Digital subtraction angiography (DSA) examination identified a basilar artery dissection indicating the delayed image of distal basilar artery and the stratification and retention of contrast agent (arrow).

Magnetic resonance imaging (MRI) and MRA demonstrated a VBD compressing the right ventrolateral region of brainstem and the trigeminal nerve root entry zone (REZ) (Figures 1A,B). The contrast-enhanced MRI revealed a significant expansion of the basilar artery (Figure 1C) and the widest part of the basilar artery was about 12-mm-diameter. Lacunar cerebral infarction was found in bilateral basal ganglia, left thalamus and right periventricular area, and hemosiderin deposition was found in right cerebellar hemisphere. The remainder of the brainstem, brain parenchyma, and cranial nerves appeared normal. Subsequent digital subtraction angiography (DSA) examination identified a basilar artery dissection indicating the delayed image of distal basilar artery and the stratification and retention of contrast agent (Figure 2). The patient underwent partial basilar dissecting aneurysm embolization based on above evidences.

### Follow-Up and Evaluation of Treatment Outcome

Immediately after surgery, pain attacks had adequate initial relief (barrow neurological institute, BNI Grades IIIa). The patient was discharged on postoperative day 7 without occurrence of other adverse events and continuous to take carbamazepine, which were stopped progressively eventually. The pain disappeared completely and did not need any more medications 6 months later. Three years after surgery, he had experienced no recurrence of the right facial pain. Physical examination including a neurological exam was normal. The final clinical diagnosis was VBD and basilar artery dissection.

## Discussion

The etiology of TN is mostly related to vascular compression of the ipsilateral trigeminal nerve, and the basilar artery is involved more frequently than the vertebral artery. VBD is an unusual cause of TN associated to vascular compression due to the expansion, elongation, and tortuosity of vertebrobasilar artery (5, 6). The direct vascular compression can lead to the dysfunction of cranial nerves (7). The local nerve demyelination and axonopathy due to the compression, can result in ephaptic transmission between the severely injured myelinated fibers and smaller unmyelinated pain fibers (8). For such patients, a burst of spontaneous firing is induced by a minor stimulation (such as a light touch) of the myelinated fibers, which is a severe pain that lasts for seconds or minutes (9, 10). Although this is rare, sometimes an ectatic vertebrobasilar vessel can be the only cause of TN. Compared with the other patients with TN, the patients with TN caused by vertebrobasilar compressions was older, more men, and more likely to suffer from hypertension (11).

The pathological feature of VBD was related to a structural arterial wall defect of the internal elastic lamina and thinning of the media secondary to smooth muscle atrophy (12). The uncontrolled hypertension and atherosclerosis have been proposed to be the pathogenic factors for the degeneration of vascular wall. And the autoimmune and inflammation mechanisms are also considered to be the risk factors of VBD (13, 14). Therefore, the inflammatory cytokines may accelerate the fracture or destruction of the internal elastic lamina of vessel wall, leading to further artery extension. The extension of vertebrobasilar is also associated with the instability causing the advancement of the vertebrobasilar dissection (15). And part of the etiologies of VBD described above are also the determinants of arterial dissection. Additionally, it has been reported that fusiform aneurysm is an artery dissection characterized by balloon inflation around the entire vessel wall for a short segment (16). The concept of fusiform aneurysm is similar to the definition of dolichoectasia. There is a connection between the two diseases. The abnormal hemodynamic stress plays an important role in the evolvement of arterial dissection and intramural hematoma. Importantly, the development of intramural hematoma is also a vital step in the pathological progression of dolichoectasia (17). Thus, vertebrobasilar artery dissection may also contribute to the exacerbation of VBD (13, 18). When the initial artery diameter is larger than 10 mm, alternative treatments like endovascular procedures and antihypertensive therapy should be considered (19).

Although TN caused by VBD is rare and the strategies for the management of VBD are systematized, patients with VBD should be given more attention than those without. The experienced radiologist, neurosurgeon and the necessary neuroimaging examinations are needed to identify the presence of arterial dissection.

## Ethics Statement

This study was approved by the Ethics Committee of the First Affiliated Hospital of Zhengzhou University (Number: KW-2018-LW-006). Written informed consent was obtained from the participant for the publication of this case report.

## Author Contributions

All authors were involved in the data collection and the preparation of the manuscript, drafted and revised the manuscript, and approved it for publication.

### Conflict of Interest Statement

The authors declare that the research was conducted in the absence of any commercial or financial relationships that could be construed as a potential conflict of interest.
